# 血液病患者合并耐碳青霉烯类肠杆菌科细菌血流感染的临床特点及预后分析

**DOI:** 10.3760/cma.j.cn121090-20240822-00315

**Published:** 2024-11

**Authors:** 利宁 张, 玉青 崔, 青松 林, 春晖 徐, 佳丽 孙, 易耕 曹, 文彬 曹, 晨 梁, 欣 陈, 卫华 翟, 巧玲 马, 荣莉 张, 嘉璘 魏, 栋林 杨, 爱明 庞, 祎 何, 尔烈 姜, 明哲 韩, 四洲 冯

**Affiliations:** 1 中国医学科学院血液病医院（中国医学科学院血液学研究所），血液与健康全国重点实验室，国家血液系统疾病临床医学研究中心，细胞生态海河实验室，天津 300020 State Key Laboratory of Experimental Hematology, National Clinical Research Center for Blood Diseases, Haihe Laboratory of Cell Ecosystem, Institute of Hematology & Blood Diseases Hospital, Chinese Academy of Medical Sciences & Peking Union Medical College, Tianjin 300020, China; 2 天津医学健康研究院，天津 301600 Tianjin Institutes of Health Science, Tianjin 30l600, China

**Keywords:** 血液病, 耐碳青霉烯类肠杆菌科细菌, 血流感染, Hematologic disease, Carbapenem-resistant Enterobacteriaceae, Bloodstream infection

## Abstract

**目的:**

分析血液病患者合并耐碳青霉烯类肠杆菌科细菌（CRE）血流感染的临床及分子学特征，探讨预后危险因素。

**方法:**

对中国医学科学院血液病医院2015年1月至2022年12月发生CRE血流感染的血液病患者进行回顾性研究，分析临床特点、碳青霉烯酶检测结果、抗感染治疗及转归。

**结果:**

共120例患者发生CRE血流感染。大肠埃希菌最多见（58/120，48.3％），其次为肺炎克雷伯菌（52/120，43.3％）。93株CRE进行了碳青霉烯酶检测，其中产碳青霉烯酶CRE共75株（金属酶51株、丝氨酸酶24株）。患者感染后30 d死亡率为24.2％（29/120）。单因素分析显示，接受含头孢他啶-阿维巴坦治疗患者的死亡率明显低于接受其他抗菌药物治疗患者（7.8％对36.2％，*P*<0.001）。此外，在感染发生24 h内接受有效抗感染治疗可显著降低死亡率（15.0％对33.3％，*P*＝0.019）。而合并CRE定植患者，在12 h、24 h内接受有效抗感染治疗的比例明显高于无定植患者（12 h：34.1％对14.5％，*P*＝0.012；24 h：65.9％对40.8％，*P*＝0.008）。多因素分析发现，合并感染性休克（*HR*＝24.436，95％ *CI* 4.148～143.966，*P*<0.001）和肺感染（*HR*＝9.346，95％ *CI* 2.718～32.140，*P*<0.001）是患者30 d内死亡的独立危险因素。而24 h内接受有效抗感染治疗（*HR*＝0.225，95％ *CI* 0.059～0.851，*P*＝0.028）以及接受含头孢他啶-阿维巴坦的治疗（*HR*＝0.082，95％ *CI* 0.018～0.362，*P*＝0.001）可显著降低死亡率。

**结论:**

血液病患者合并CRE血流感染预后差，及早启动有效抗感染治疗以及应用含头孢他啶-阿维巴坦的方案可提高生存率、改善预后。

## Abstract (en)

**Objective:**

This study aimed to analyze the clinical and molecular characteristics of carbapenem-resistant Enterobacteriaceae（CRE）bloodstream infection（BSI）in patients with hematological diseases and to explore prognostic risk factors.

**Methods:**

This retrospective study included patients with hematologic diseases with CRE BSI at the Institute of Hematology and Blood Diseases Hospital from January 2015 to December 2022. The clinical features, carbapenemase test results, antimicrobial treatments, and outcomes were analyzed.

**Results:**

A total of 120 patients developed CRE BSI. Escherichia coli（58/120, 48.3％）was the most prevalent Enterobacteriaceae, followed by Klebsiella pneumoniae（52/120, 43.3％）. A total of 93 CRE strains were tested for carbapenemase, of which 75 strains produced carbapenemase（metalloenzyme: 51 strains; serine enzyme: 24 strains）. The 30-day mortality rate after BSI was 24.2％（29/120）. Univariate analysis revealed significantly lower mortality in patients treated with the ceftazidime-avibactam-containing regimen than in those treated with other antibiotics（7.8％ *vs* 36.2％, *P*<0.001）. Moreover, initiating active therapy within 24 h of BSI onset significantly reduced mortality（15.0％ *vs* 33.3％, *P*＝0.019）. The proportion of patients with CRE colonization receiving active therapy within 12 and 24 h was significantly higher compared with patients without colonization（12 h: 14.5％ *vs* 34.1％, *P*＝0.012; 24 h: 40.8％ *vs* 65.9％, *P*＝0.008）. Multivariate analysis revealed that septic shock（*HR*＝24.436, 95％ *CI* 4.148–143.966, *P*<0.001）and pulmonary infection（*HR*＝9.346, 95％ *CI* 2.718–32.140, *P*<0.001）were independent risk factors for death within 30 days. Appropriate therapy was initiated within 24 h（*HR*＝0.225, 95％ *CI* 0.059–0.851, *P*＝0.028）, and treatment with the ceftazidime-avibactam-containing regimen（*HR*＝0.082, 95％ *CI* 0.018–0.362, *P*＝0.001）significantly reduced mortality.

**Conclusion:**

The prognosis of CRE BSI in patients with hematological diseases is poor. Timely, appropriate therapy and receipt of a ceftazidime-avibactam-containing regimen can improve survival and prognosis.

肠杆菌科细菌是临床最常见的革兰阴性菌，而随着碳青霉烯类药物的广泛应用，碳青霉烯类耐药的肠杆菌科细菌（carbapenem-resistant Enterobacterales, CRE）感染的发生率逐年增加，对全球公共卫生安全构成重大威胁[1]。血液病患者由于免疫功能低下、粒细胞缺乏（粒缺）、放化疗相关黏膜炎、接受造血干细胞移植等，是发生CRE感染的高危人群[2]。由于临床可选的抗菌药物非常有限，CRE感染治疗难度大，死亡率通常较高，尤其是血流感染。本研究对120例发生CRE血流感染的血液病患者进行回顾性研究，分析其临床及分子学特征，探索预后影响因素和合适的抗菌药物选择。

## 病例与方法

1. 病例资料：回顾性分析2015年1月至2022年12月在中国医学科学院血液病医院发生CRE血流感染的血液病患者的临床资料。排除标准：①合并其他病原菌的混合血流感染；②在获得血培养阳性结果之前患者出院。对于发生多次CRE血流感染的患者，仅分析首次感染。共入组120例CRE血流感染患者，收集以下数据：基本临床资料、血液系统疾病及治疗、CRE病原菌及来源、药物敏感性分析及碳青霉烯酶检测结果、抗感染治疗及结局等。

2. 定义：CRE定义为对任一碳青霉烯类药物耐药［亚胺培南、美罗培南、多利培南的最低抑菌浓度（MIC）≥4 mg/L，厄他培南MIC ≥2 mg/L］或产碳青霉烯酶。如果是对亚胺培南天然耐药的细菌（如摩氏摩根菌变形杆菌属、普罗威登斯菌属），必须对其他碳青霉烯类药物（如美罗培南、厄他培南、多利培南）耐药[3]。根据美国疾病控制与预防中心的定义确定血流感染的疑似来源[4]。单药治疗定义为仅包含一种具有体外活性的抗菌药物的治疗；联合治疗定义为包含两种或两种以上具有体外活性的抗菌药物的治疗。纳入分析的抗菌药物需至少使用48 h。粒缺定义为患者外周血中性粒细胞绝对值（ANC）<0.5×10^9^/L。

3. 微生物学检测：采用全自动血培养仪（美国BD公司产品）进行血培养，采用VITEK2 compact进行菌株的鉴定和药物敏感性分析。碳青霉烯酶表型检测采用改良碳青霉烯灭活试验（mCIM）、EDTA-碳青霉烯灭活试验（eCIM）。应用PCR技术检测常见的碳青霉烯酶基因，包括blaKPC、blaNDM、blaOXA-48、blaIMP、blaVIM和blaOXA-23。

4. 抗感染治疗方案：51例患者接受含头孢他啶-阿维巴坦的治疗，其中43例联合氨曲南。69例患者接受不含头孢他啶-阿维巴坦的治疗，其中单药治疗共27例，抗菌药物包括替加环素、氨基糖苷类、多黏菌素。联合治疗共42例，抗菌药物包括替加环素、氨基糖苷类、多黏菌素、喹诺酮类、磷霉素，其中替加环素+氨基糖苷类±喹诺酮类（69.0％，29/42）为最常用的联合治疗方案。

5. 统计学处理：采用SPSS 22.0进行统计学分析。连续性变量间的差异比较采用独立样本*t*检验（符合正态分布）或Mann-Whitney *U*检验（不符合正态分布）；分类变量间的差异性比较采用卡方检验或Fisher精确检验。多因素分析使用Logistic回归。双侧*P*<0.05为差异有统计学意义。

## 结果

1. 临床特征：120例CRE血流感染患者中，男74例，女46例，中位年龄38（26，49）岁。血液系统疾病分布：急性髓系白血病52例（43.3％）、急性淋巴细胞白血病29例（24.2％）、再生障碍性贫血16例（13.3％）、骨髓增生异常综合征11例（9.2％）、其他血液系统疾病12例（10.0％）。发生血流感染时，94.2％（113/120）的患者处于粒缺期。原发血流感染共61例（50.8％），继发血流感染共59例（49.2％），原发感染灶包括肛周（22例）、肠道（17例）、肺部（7例）、口咽部（7例）、皮肤软组织（5例）、齿龈（1例）。在既往3个月内，44例患者合并CRE定植，其中1例为咽部定植，6例为咽部、肛周同时定植，余37例均为肛周定植。在CRE血流感染发生12、24和48 h内分别有26例（21.7％）、60例（50.0％）、108例（90.0％）患者接受有效抗感染治疗。既往3个月内合并CRE定植的患者，在血流感染发生12 h、24 h内接受有效抗感染治疗的比例显著高于无定植患者（12 h：34.1％对14.5％，*P*＝0.012；24 h：65.9％对40.8％，*P*＝0.008）。

2. 病原菌及碳青霉烯酶检测结果：在120株CRE中，大肠埃希菌最多见（58株，48.3％），其次为肺炎克雷伯菌（52株，43.3％）、阴沟肠杆菌（8株，6.7％）、产气肠杆菌（1株，0.8％）和植生拉乌尔菌（1株，0.8％）。这些菌株对替加环素的敏感性最高，为76.7％；其次为阿米卡星，敏感性为69.2％。而对其他种类抗生素（包括喹诺酮类、头孢菌素类、庆大霉素、妥布霉素等）表现出高度耐药性。共93株CRE进行了碳青霉烯酶表型检测，其中产碳青霉烯酶的CRE（CP-CRE）共75株（金属酶51株；丝氨酸酶24株），非CP-CRE共18株。54株CP-CRE进行了碳青霉烯酶基因检测，其中产NDM酶37株、产KPC酶16株、产IMP酶1株。在产NDM酶的CRE菌株中，1株同时产OXA-48酶。在24株产碳青霉烯酶肺炎克雷伯菌（CRKp）中，最常见的碳青霉烯酶的是KPC酶（15株），其次是NDM酶（9株）。而在25株产碳青霉烯酶大肠埃希菌中，所有菌株均产NDM酶（表1）。

**表1:** 耐碳青霉烯类肠杆菌科细菌（CRE）血流感染患者菌株的碳青霉烯酶表型及基因检测结果

	总体（93株）	大肠埃希菌（42株）	肺炎克雷伯菌（43株）	其他病原菌（8株）
表型检测［例（％）］				
CP-CRE	75（80.6）	35（83.3）	35（81.4）	5（62.5）
非CP-CRE	18（19.4）	7（16.7）	8（18.6）	3（37.5）
基因检测［阳性例数/总例数（％）］				
NDM	36/54（66.7）	25/25（100.0）	9/24（37.5）	3/5（60.0）
KPC	16/54（29.6）	0/25（0）	15/24（62.5）	1/5（20.0）
IMP	1/54（1.9）	0/25（0）	0/24（0）	1/5（20.0）
OXA-48	1/54（1.9）	1/25（4.0）^a^	0/24（0）	0/5（0）
VIM	0/54（0）	0/25（0）	0/24（0）	0/5（0）
OXA-23	0/54（0）	0/25（0）	0/24（0）	0/5（0）

**注** CP-CRE：产碳青霉烯酶的CRE；^a^该CRE菌株同时产NDM酶

3. 血流感染转归及预后危险因素分析：共29例（24.2％）患者在发生血流感染30 d内死亡。单因素分析见表2，合并感染性休克（63.2％对16.8％，*P*<0.001）、肺感染（50.0％对13.1％，*P*<0.001）的患者30 d死亡率明显增加。此外，粒缺持续时间≥14 d的患者死亡率更高（30.1％对10.8％，*P*＝0.022）。而在血流感染发生24 h内接受有效抗感染治疗可显著降低死亡率（15.0％对33.3％，*P*＝0.019）。此外，含头孢他啶-阿维巴坦的方案明显优于不含头孢他啶-阿维巴坦的方案，两组30 d死亡率分别为7.8％、36.2％（*P*<0.001）。我们还比较了CP-CRE与非CP-CRE、年龄、性别等对预后的影响，差异均无统计学意义（均*P*>0.05）。

**表2:** 影响患者CRE血流感染后30 d死亡的单因素分析

临床特征	所有患者（120例）	感染后30 d死亡		*P*值
		是（29例）	否（91例）	
年龄［岁，*M*（*IQR*）］	38（26，49）	39（20，50）	38（27，49）	0.609
男性［例（％）］	74（61.7）	18（24.3）	56（75.7）	0.959
血液系统疾病［例（％）］				0.411
急性髓系白血病	52（43.3）	11（21.2）	41（78.8）	
急性淋巴细胞白血病	29（24.2）	6（20.7）	23（79.3）	
再生障碍性贫血	16（13.3）	7（43.8）	9（56.3）	
骨髓增生异常综合征	11（9.2）	3（27.3）	8（72.7）	
其他	12（10.0）	2（16.7）	10（83.3）	
接受造血干细胞移植［例（％）］	29（24.2）	4（13.8）	25（86.2）	0.134
粒缺持续时间≥14 d［例（％）］	83（69.2）	25（30.1）	58（69.9）	0.022
CRE菌株［例（％）］				0.101
大肠埃希菌	58（48.3）	13（22.4）	45（77.6）	
肺炎克雷伯菌	52（43.3）	16（30.8）	36（69.2）	
其他	10（8.3）	0（0）	10（100.0）	
CP-CRE［例（％）］	75（80.6）	14（18.7）	61（81.3）	0.389
合并肺感染［例（％）］	36（30.0）	18（50.0）	18（50.0）	<0.001
合并感染性休克［例（％）］	19（15.8）	12（63.2）	7（36.8）	<0.001
12 h内接受有效抗感染治疗［例（％）］	26（21.7）	3（11.5）	23（88.5）	0.089
24 h内接受有效抗感染治疗［例（％）］	60（50.0）	9（15.0）	51（85.0）	0.019
抗感染治疗方案［例（％）］				<0.001
含CAZ-AVI的治疗	51（42.5）	4（7.8）	47（92.2）	
不含CAZ-AVI的治疗	69（57.5）	25（36.2）	44（63.8）	

**注** CRE：耐碳青霉烯类肠杆菌科细菌；粒缺：粒细胞缺乏；CP-CRE：产碳青霉烯酶的耐碳青霉烯类肠杆菌科细菌；CAZ-AVI：头孢他啶-阿维巴坦

将单因素分析中*P*<0.05的变量纳入多因素Logistic回归分析，结果见表3。合并感染性休克（*HR*＝24.436，95％ *CI* 4.148～143.966，*P*<0.001）和肺感染（*HR*＝9.346，95％ *CI* 2.718～32.140，*P*<0.001）是血液病患者CRE血流感染30 d内死亡的独立危险因素。而24 h内接受有效抗感染治疗（*HR*＝0.225，95％ *CI* 0.059～0.851，*P*＝0.028）以及接受含头孢他啶-阿维巴坦的治疗（*HR*＝0.082，95％ *CI* 0.018～0.362，*P*＝0.001）可显著降低死亡率，改善预后。

**表3:** 影响患者CRE血流感染30 d死亡的多因素分析

影响因素	*HR*	95％ *CI*	*P*值
肺感染	9.346	2.718～32.140	<0.001
感染性休克	24.436	4.148～143.966	<0.001
24 h内接受有效抗感染治疗	0.225	0.059～0.851	0.028
含头孢他啶-阿维巴坦的治疗	0.082	0.018～0.362	0.001
粒缺持续时间≥14 d	2.426	0.561～10.489	0.235

**注** CRE：耐碳青霉烯类肠杆菌科细菌；粒缺：粒细胞缺乏

## 讨论

血液病患者是CRE感染的高危人群，而且感染相关死亡率较高，尤其是CRE血流感染[5]–[6]。我们的研究共纳入120例发生CRE血流感染的血液病患者，94.2％的患者处于粒缺期，且69.2％的患者粒缺持续时间≥14 d。患者感染后30 d死亡率为24.2％（29/120）。本研究显示，及早启动有效的抗感染治疗以及应用含头孢他啶-阿维巴坦的方案是降低患者死亡率、改善预后的关键。

本研究中，大肠埃希菌（48.3％）是最常见的CRE血流感染菌株，其次是肺炎克雷伯菌（43.3％）。而在欧美国家及国内其他中心，肺炎克雷伯菌是主要致病菌（70％～85％），其次为大肠埃希菌[7]–[9]。CRE绝大多数产碳青霉烯酶，对碳青霉烯酶表型或基因型检测具有重要意义[10]。国内的多个研究[6],[9],[11]均报道产KPC酶和NDM酶的CRE菌株最多见，其中肺炎克雷伯菌以KPC酶为主，而大肠埃希菌以NDM酶为主。本研究中，CP-CRE占80.6％（75/93），其中金属酶占54.8％（51/93），丝氨酸酶占25.8％（24/93）。对碳青霉烯酶进行基因检测，我们同样发现NDM酶和KPC酶最常见。但值得注意的是，在产碳青霉烯酶的大肠埃希菌中，所有菌株均产NDM酶。而在CRKp中，虽然仍以KPC酶为主（62.5％），但NDM酶也占了相对较高的比例（37.5％）。可以看出，我院CRE血流感染的菌株及碳青霉烯酶分布，与其他中心存在差异，其中产金属酶的CRE比例明显偏高。

对于CRE血流感染，多数研究[12]–[14]均证实及早启动有效的抗感染治疗是改善患者预后的关键。本研究也发现，血流感染发生24 h内接受有效抗CRE治疗患者的死亡率明显低于24 h内未覆盖CRE患者（15.0％对33.3％，*P*＝0.019）。进一步分析提示，早期有效抗感染治疗在既往合并CRE定植的患者中的比例更高。既往徐春晖等[15]分析了我院2 914例血液病患者的肛周拭子培养结果，其中74例检出CRE，13例后期发生CRE血流感染（17.6％），且肛周拭子与血液中分离CRE的药敏一致性较高。因此，对于血液病患者，尤其是高危感染患者，CRE主动筛查具有重要意义[16]。对于合并CRE定植的患者，在发生CRE血流感染时可更早识别，而经验性覆盖CRE治疗时可参考定植菌耐药情况。因此，加强血液病患者CRE筛查，对于早期启动有效抗CRE治疗有重要作用。

头孢他啶-阿维巴坦是一种新型β-内酰胺酶抑制剂，多项研究[17]–[21]证实头孢他啶-阿维巴坦可显著降低CRE感染的病死率，且不良反应发生率低，优于其他抗CRE治疗方案。目前对于非产金属酶的CRE感染，建议优先选择头孢他啶-阿维巴坦治疗[10],[16]。而对于产金属酶的CRE，氨曲南不易被金属酶水解，头孢他啶-阿维巴坦与氨曲南联合具有良好的体外协同活性，推荐用于产金属酶的CRE感染[10]。一项前瞻性研究[22]比较了头孢他啶-阿维巴坦联合氨曲南（52例）和其他抗菌药物（50例）治疗产金属酶的CRE血流感染的疗效，结果发现头孢他啶-阿维巴坦联合氨曲南治疗组30 d死亡率显著降低（19.2％对44％，*P*＝0.007）。本研究中，51例患者接受含头孢他啶-阿维巴坦的治疗，余69例接受其他抗菌药物单药或联合治疗。同样地，应用头孢他啶-阿维巴坦治疗是改善患者预后的独立因素（*HR*＝0.082，95％ *CI* 0.018～0.362，*P*＝0.001）。此外，在该组患者中，84.3％同时联合氨曲南，主要是考虑我院产金属酶的CRE比例明显较高。而对于大肠埃希菌，鉴于检测的碳青霉烯酶均为NDM酶，因此如果选择头孢他啶-阿维巴坦，应同时联合氨曲南。

综上所述，血液病患者合并CRE血流感染预后差，其中合并感染性休克或肺感染的患者病死率更高。在感染发生24 h内接受有效抗感染治疗以及应用含头孢他啶-阿维巴坦的方案是降低患者死亡率、改善预后的关键。CRE主动筛查意义重大，有助于CRE血流感染的早期识别及治疗。由于各中心碳青霉烯酶分布存在差异，对CRE菌株进行碳青霉烯酶检测具有重要意义，可指导抗菌药物选择，优化抗CRE治疗方案。
